# Morphological alterations in the caudate, putamen, pallidum, and thalamus in Parkinson's disease

**DOI:** 10.3389/fnins.2015.00101

**Published:** 2015-03-31

**Authors:** Amanmeet Garg, Silke Appel-Cresswell, Karteek Popuri, Martin J. McKeown, Mirza Faisal Beg

**Affiliations:** ^1^Medical Image Analysis Laboratory, School of Engineering Science, Simon Fraser UniversityBurnaby, BC, Canada; ^2^Neurology, Pacific Parkinson's Research Center, University of British ColumbiaVancouver, BC, Canada

**Keywords:** LDDMM, shape analysis, surface displacement, Parkinson's disease, prediction, brain MRI

## Abstract

Like many neurodegenerative diseases, the clinical symptoms of Parkinsons disease (PD) do not manifest until significant progression of the disease has already taken place, motivating the need for sensitive biomarkers of the disease. While structural imaging is a potentially attractive method due to its widespread availability and non-invasive nature, global morphometric measures (e.g., volume) have proven insensitive to subtle disease change. Here we use individual surface displacements from deformations of an average surface model to capture disease related changes in shape of the subcortical structures in PD. Data were obtained from both the University of British Columbia (*UBC*) [*n* = 54 healthy controls (HC) and *n* = 55 Parkinsons disease (PD) patients] and the publicly available Parkinsons Progression Markers Initiative (*PPMI*) [*n* = 137 (HC) and *n* = 189 (PD)] database. A high dimensional non-rigid registration algorithm was used to register target segmentation labels (caudate, putamen, pallidum, and thalamus) to a set of segmentation labels defined on the average-template. The vertex-wise surface displacements were significantly different between PD and HC in thalamic and caudate structures. However, overall displacements did not correlate with disease severity, as assessed by the Unified Parkinson's Disease Rating Scale (UPDRS). The results from this study suggest disease-relevant shape abnormalities can be robustly detected in subcortical structures in PD. Future studies will be required to determine if shape changes in subcortical structures are seen in the prodromal phases of the disease.

## 1. Introduction

Parkinson's disease (PD) is the second most common age related neurodegenerative disorder after Alzheimers disease (de Lau and Breteler, 2006). Routine clinical MRI is rarely used in diagnosis, and is often used only to rule out other conditions that may mimic PD (e.g., vascular Parkinsonism). Although there are no gross structural abnormalities seen in PD, the use of structural MRI is still potentially attractive as a biomarker because of the ubiquitous nature of the technology. Several studies have suggested subtle morphological alterations such as atrophy in the putamen and/or caudate (Schulz et al., 1999; Ghaemi et al., 2002; Krabbe et al., 2005; Pitcher et al., 2012). Most of these studies have looked at overall volume as a measure of atrophy as it easy to measure, invariant to position of the subject in the scanner, and, if appropriately normalized, directly comparable across subjects. However, in some structures such as the thalamus, volume may actually increase as a compensatory mechanism when cortical regions are damaged (Pol and van der Flier, 2000), complicating overall volume as a marker of disease progression. This may be why the thalami undergo significant shape change with PD, even when no significant difference in volume can be detected (McKeown et al., 2008), presumably on the basis of specific nuclei being affected and/or compensatory hypertrophy of other regions.

Commonly-used brain morphometric analysis methods to assess progression of neurodegenerative disease can be categorized into three general types: voxel-based, surface-based, and deformation-based methods. By far, the majority of morphometry studies to date have been based on voxel-based morphometry (VBM), because of its conceptual simplicity and widespread availability of suitable software. In VBM, subjects brain images are registered to a common template image, and statistical analyses are performed on a voxel-by-voxel basis on the registered subject brain images. This technique has demonstrated cortical loss in some brain areas in PD (Burton et al., 2005; Nagano-Saito et al., 2005) and in other PD-related diseases (e.g., Multi system atrophy) (Paviour et al., 2006; Brenneis et al., 2007; Tzarouchi et al., 2010). Nevertheless, there is widespread recognition of the limitations of VBM: it tends to find focal changes as opposed to more spatially distributed changes (Davatzikos, 2004), and may be insensitive to subtle morphological alterations (Bergouignan et al., 2009).

An alternative approach is to use surface-based morphometry (SBM) for measuring shape changes. In SBM, a surface representation of a structural boundary is investigated rather than at the level of the individual voxels. Using such a method, PD related shape and volume changes in the hippocampus, caudate and ventricles can be detected (Apostolova et al., 2010). In a similar approach, deformation-based morphometry (DBM), the deformation fields obtained from one-to-one non-rigid registrations are analyzed in place of the final registered images (Duchesne et al., 2009). In our work, we use a SBM method where we model the change in shape as the deformation of a template surface to the individual target surfaces for the disease and control groups. The surface displacement metric obtained as a signed normal component of the displacement vector (from template to target surface) at each vertex on the template surface captures the deformation information. This metric directly models deformation on the surface of the template.

Here we present a SBM method that incorporates anatomically-defined regions of interest (ROIs). Accurate segmentation labels for subcortical ROIs were obtained via an automated registration based segmentation process (Khan et al., 2008). These labels were then registered to a prototype via a non-linear registration algorithm (Beg et al., 2005). The template for this study was generated via an alternating registration and averaging process (Khan and Beg, 2009), that encapsulates information from the entire cohort and thus with minimal bias to data from a specific subject. Given a mean template, the surface displacement obtained from taking the difference in coordinates between the reference (template) and the deformed surfaces on a vertex-by vertex basis represent a feature for subsequent classification. Based on prior work (McKeown et al., 2008; Apostolova et al., 2010) we study the change in shape of caudate, thalamus, putamen, and pallidum structures due to PD. We processed the imaging data from two cohorts with PD patients and healthy control groups, and analyzed the surface displacement feature for the group level difference in the features.

The rest of the paper is organized as follows: in Section 2 the data and processing methods are introduced, followed by the results of experiments in Section 3. The results are further discussed and concluded in Section 4.

## 2. Materials and methods

In this section we discuss the methods for extraction of shape features from the MRI data for the individual subcortical ROIs. The feature extraction process consists of a number of steps applied sequentially to perform the tasks of data pre-processing, construction of a prototype, diffeomorphic registration followed by final feature extraction. These features are then tested for differences between the PD and HC groups. The following sub-sections provide a description of these steps in detail.

### 2.1. Subjects and scans

#### 2.1.1. University of British Columbia (*UBC*) dataset

Data were taken from 55 non-demented PD subjects and 54 healthy subjects seen at the *UBC Pacific Parkinson's Research Center*. Table 1 gives the clinical and demographic details of the PD and control groups. Written informed consent was obtained from all subjects prior to participation in the study. This study was approved by the Clinical Research Ethics Board of the University of British Columbia and conforms to the Declaration of Helsinki.

**Table 1:** **Demographics for the data in the *UBC* dataset**.

**Variable**	**Healthy control**	**PD subjects**
Number	54	55
Age	46.16 ± 16.80	64 ± 8.11
Gender	23M/25F	36M/19F
UPDRS	N.A.	25.89 ± 11.02

Patients were examined after overnight medication withdrawal with >12 h for L-dopa and >18 h for dopamine agonists. Exclusion criteria included atypical parkinson's (cerebellar ataxia, prominent dementia, early postural instability), symmetrical onset of symptoms, evidence of severe memory impairment or signs of dementia [Montreal Cognitive Assessment (MoCA) scores < 24] or a history of cerebrovascular disease or other neurological disorders. The Unified Parkinson's Disease Rating Scale (UPDRS) motor scale data were recorded and used in further analysis.

Subjects were scanned using a Philips Achieva 3.0 T scanner (Philips, Best, The Netherlands), with a 8-channel head coil. A memory foam pillow was used to minimize head motion. The acquisition parameters for the 3D T1-TFE sequence were as follows: 170 axial slices, repetition time = 7.7 ms, echo time = 3.6 ms, flip angle 8°, field of view 256 × 200 mm, acquired matrix size 256 × 200, and voxel size 1 × 1 × 1 mm^3^.

#### 2.1.2. Parkinson's progression marker initiative (*PPMI*) cohort

The data available under the *PPMI* project was obtained from the LONI Image data archive (https://ida.loni.usc.edu). The data acquisition details are available on the website for the PPMI project and can be obtained from the url (http://www.ppmi-info.org/). The data from the baseline visit was selected for the analysis. We randomly chose a subset of subjects so that they would be age-matched with other subject groups. The demographic details for this subset are presented in the Table 2.

**Table 2:** **Demographics for the data in the *PPMI* dataset**.

**Variable**	**Healthy control**	**PD patients**
Number	137	189
Age	63.85 ± 7.46	68.02 ± 4.77
Gender	75M/62F	115M/74F
UPDRS	N.A.	33.62 ± 13.36

The *PPMI* cohort is a multicenter study where multiple scanners and imaging protocols are used. An example protocol for a MPRAGE sequence as used in the study is as follows: Siemens Magnetom TrioTim sungo MR B17 3T scanner (Siemens, Germany). T1 weighted images were acquired with the MPRAGE sequence with the following parameters: 176 axial slices, repetition time = 2300 ms, echo time = 2.98 ms, flip angle = 9°, field of view = 256 mm, acquired matrix size = 256 × 200, and voxel size 1 × 1 × 1 mm^3^. Some other scanners used in the study include GE Signa 3.0T, GE Discovery 3.0T (G.E., USA), Philips Acheiva 1.5T (Philips, Netherlands), Siemens TrioTim 3.0T, Siemens Symphony 1.5T, Siemens Verio 3.0T, Siemens Espree 1.5T (Siemens, Germany). Extensive pre-processing and data normalization was performed by the members of the PPMI-core group prior to release of the data for public use. For detailed image acquisition protocols and scanner specific preprocessing the reader is referred to the website of the PPMI cohort at www.ppmi-info.org

### 2.2. Data analysis process

**Figure 1:**
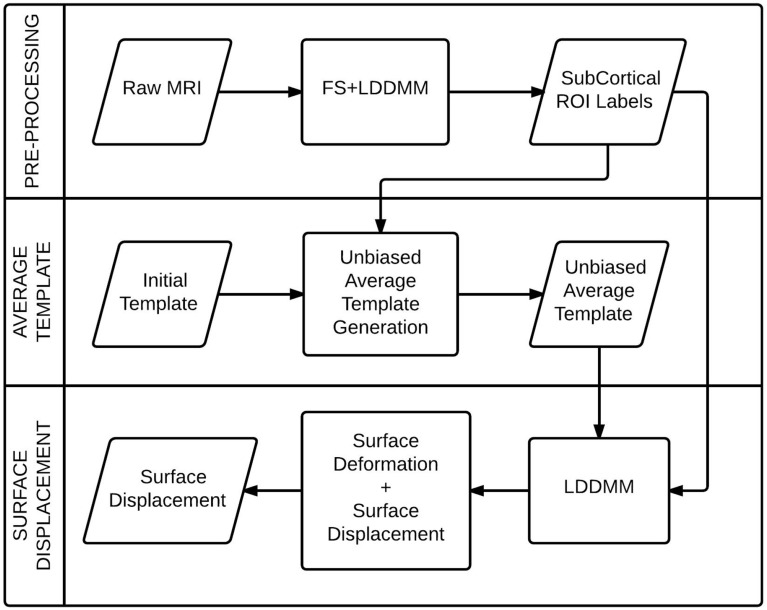
**A process flow diagram for the computation of the surface displacement feature from the raw MRI data**.

In this section, we present the details of the image analysis steps that was used to obtain the surface displacement data for individual subcortical structures from the raw MRI data. We briefly describe the registration algorithm, followed by the methods for segmentation of anatomical structures, prototype creation and surface displacement computation. The process flow diagram (Figure 1) illustrates the stages of the process.

#### 2.2.1. Large-deformation diffeomorphic metric mapping (LDDMM)

Diffeomorphic registration methods are desirable in processing of medical imaging data because of their inherent smoothness and ability to model large and small displacements. Here we briefly describe the LDDMM (Beg et al., 2005) process, which generates a diffeomorphic transformation by minimizing the difference between the source and transformed target images.

Let us define Ω ⊂ ℝ^3^ as the coordinate space of the source image, and *G*:Ω ↔ Ω as the set of diffeomorphic transformations on Ω. The LDDMM algorithm seeks a geodesic ϕ : [0, 1] → *G* where each point ϕ_*t*_ ∈ *G, t* ∈ [0, 1] is a diffeomorphism on the domain Ω. Then the source image *I*_0_ evolves along the path to the target image *I*_1_ according to ϕ_*t*_*I*_0_ = *I*_0_ ◦ ϕ^−1^_*t*_. At the endpoint *t* = 1, the source *I*_0_ is connected to the target image via *I*_1_ = ϕ_1_*I*_0_ = *I*_0_ ◦ ϕ^−1^_1_. The associated velocity field *v*, taken from the space of smooth velocity fields *V* on the domain Ω ⊂ ℝ^3^, is a solution to the differential equation ${\dot{\phi }}_{t}$ = *v*_*t*_ (ϕ_*t*_), *t* ∈ [0, 1] satisfying

(1) $$\begin{array}{l} \hat{v}=v:{\dot{\phi }}_{t}\\ \;=\arg\;{\text{min}}_{V_{t}(\phi_{t})}\left(\int_{0}^{1}\|v_{t}{\|}^{2}\text{d}t+\frac{1}{\sigma^{2}}\|I_{0}{}^{\circ}\phi_{1}^{-1}-I_{1}{\|}_{L^{2}}^{2}\right) \end{array}$$

By integrating the optimizer $\hat{v}$ of this cost function we get the optimal change of coordinates $\varphi =\phi_{1}^{\hat{v}}$. The superscript *v* in ϕ^*v*^ is used to explicitly denote the dependence of ϕ on the associated velocity field *v*. The mapping $\varphi =\phi_{1}^{\hat{v}}$ is guaranteed to be a diffeomorphism by enforcing sufficient smoothness on the elements of *V*. We do this by defining a norm on *V* through a 3 × 3 differential operator *L* of the type *L* = (αΔ + γ)^α^*I*_3×3_ where α > 1.5 in 3D space such that ‖*f*‖_*V*_ = ‖*Lf*‖_*L*_2__, and ‖ · ‖_*L*_2__ is the standard *L*_2_ norm for the square integrable functions defined on Ω. The gradient of the cost function (Equation 1) is given by the following Freche derivative in *V*:

(2) $$\nabla_{v}E_{t}=2{\hat{v}}_{t}-K\left(\frac{2}{\sigma^{2}}|D\phi_{t,1}^{\hat{v}}|\nabla J_{t}^{0}(J_{t}^{0}-J_{t}^{1})\right)$$

Where *J*^0^_*t*_ = *I*_0_ ◦ ϕ_*t*, 0_ and *J*^1^_*t*_ = *I*_1_ ◦ ϕ_*t*, 1_, |*Dg*| is the determinant of the Jacobian matrix and *K* is a compact self-adjoint operator *K* : *L*_2_ (Ω, ℝ^3^) → *V* uniquely defined by 〈*a, b*〉_*L*_2__ = 〈*K a, b*〉_*V*_ such that for any smooth vector field *f* ∈ *V, k*(*L*^†^*L*)*f* = *f* holds. Also *L*^†^ is the adjoint of *L* and the notation ϕ_*s, t*_ = ϕ_*s*_ · ϕ^−1^_*j*_ is employed. Finally the parameter $\frac{1}{\sigma^{2}}$ provides weighted optimization between the regularization and data matching components, and is chosen to be the same for all matchings.

In order to compute *v*, this variational gradient is used in the standard gradient descent procedure, yielding the update *v*^*n* + 1^ = *v^n^* − ε ∇*_v^n^_E* where *n* denotes the simulation number.

The optimal mapping ϕ_0, 1_ is used in further steps to transform the images from the source space to the target image space.

#### 2.2.2. FS + LDDMM segmentation

The FS + LDDMM segmentation steps (Khan et al., 2008) combine the probabilistic labels obtained from the FreeSurfer (FS) program along with the LDDMM registration with ground truth template data, to create accurate labels for the subcortical ROIs in the target MRI data.

Freesurfer (v4.5.0) (Fischl et al., 2002) was utilized to obtain initial segmentation of subcortical structures for each MRI image volume. With this process, non-brain tissue was removed from the images, followed by automated Talairach transformation and segmentation of caudate, pallidum, putamen, thalamus (Fischl et al., 2002; Fischl and Kouwe, 2004). Subsequently, an ROI was defined for each structure on the target and template MR images using FS labels and manual labels, respectively. These ROIs from target images were then aligned via an intensity-based affine transformation to those in the template images. A bounding box, predefined in the template space using the extent of the template FS labels plus a 12-voxel padding, was used to generate sub-volumes. These pre-processed MRI sub-volumes were then registered via the LDDMM method as described above (Section 2.2.1) to obtain the final segmentation labels.

Within the FS + LDDMM process, the LDDMM registration was performed in a multi-stage fashion, each stage using different image pairs and with each subsequent stage initialized with the velocity vector fields, of the previous stage. In the first stage, the FS labels of the hippocampus, amygdala, and lateral ventricles were used, in the second stage, Gaussian smoothed (σ = 5) MRI images were used, and in the final stage non-smoothed MRI images were used. The velocity vector fields were discretized into 5 timesteps. Finally, the atlas segmentations for each hemisphere were propagated to the target by applying the LDDMM and affine transformation, using linear interpolation to maintain precision when resampling the segmentations. The propagated segmentations from each template were fused with equal weights for each template. This resulted in a binary segmentation volume with the same dimensions and orientation as the original MRI in the native image space with each subcortical region of interest represented by a voxel intensity of 1 (intensity threshold = 127.5) and background intensity as 0.

The templates for segmentation of the MRI images in this study were obtained by manual segmentation of 6 healthy control subjects from the UBC scanner but not included in this study. The protocols for definition of structural boundaries for all the structures of interest were obtained from previously published work (Hammers et al., 2003).

#### 2.2.3. Segmentation quality check

**Figure 2:**
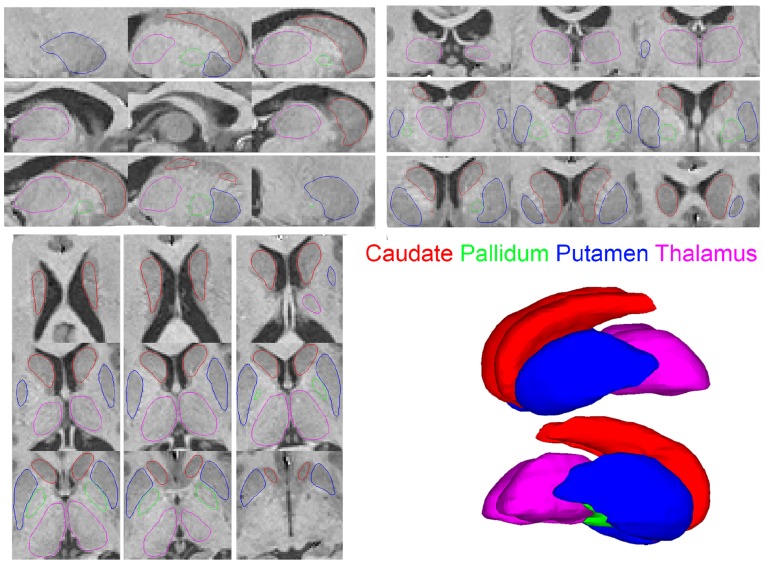
**Saggital *upper left* Coronal *upper right* Axial *lower left* views of the segmentation outlines overlayed on the corresponding MRI slices**. The 3D surface renderings show the smooth surfaces *lower right*.

The final labeling for each structure obtained from the segmentation process was checked for segmentation accuracy through an extensive quality control process. A surface model was fit for each binary segmentation volume via the marching cubes algorithm to provide a set of vertices and triangles representing the segmentation boundaries. These surfaces were overlayed on the original MRI images in the three orthogonal views to check for accuracy of segmentation labels (e.g., Figure 2). A visual verification of each such visualization was performed by an expert in neuro-anatomy. In conventional quality control approaches, the subjects with inaccurate segmentation for any structure are removed from the subsequent analysis. In our work, all subjects were found to have accurate segmentation labels and were included in the subsequent analyses. The demographics reported in Tables 1, 2 present the set of subjects with accurate and acceptable segmentation labels. The detailed visualizations for all the subjects in the analysis are available at the website (www.autobrainmapping.com).

#### 2.2.4. Unbiased average template (prototype) generation

The choice of template for registration influences the accuracy of the surface displacement data. To this end we created a “prototype” for the cohort (an average template) from the binary segmentation labels obtained for individual structures (Khan and Beg, 2009). Pre-processed data were obtained by affine alignment of each binary subcortical image to an initial template, followed by extraction of a sub-volume ROI in the prototype space. The process alternated between (1) registration (LDDMM) of individual ROIs to a template ROI and (2) computation of an average from the registered ROIs in the template space. The average computed in the previous step formed the template for registration in the next step. A healthy control subject was chosen as an initial template and the pipeline was run for three iterations to obtain the final, average unbiased template. A subset of normal subjects (*n* = 10) from the *UBC* dataset were selected for creation of the prototype.

#### 2.2.5. Surface displacement

The prototype generated above (Section 2.2.4) was used for computation of the surface displacement data. The target binary labels were pre-registered to the prototype prior to non-rigid registration. Two different approaches were considered for the pre-registration step: (1) using a 6 degrees-of-freedom (DOF) rigid transformation (2) using a 9 DOF affine transformation. The rigid pre-registration approach corrects only for the translational and rotational discrepancies between the target structures, whereas the affine approach corrects the scale discrepancy as well. Both pre-registered sets (rigid and affine) were further used in the pipeline to extract two sets of the surface displacement features.

High dimensional non-rigid registration (LDDMM) from the prototype segmentation image, *M_avg_*, to each pre-registered segmentation image *M*_*j*_ was performed to obtain the mapping ϕ_*M_avg_*, *M_j_*_ = *LDDMM*(*M_avg_*, *M*_*j*_). The injected-surface was then computed as ${\hat{S}}_{i}=\phi_{M_{avg},M_{j}}^{-1}(\bar{S})$, where each ${\hat{S}}_{i}$ has the same set of corresponding nodes, obtained from the template mesh, *S*, and thus all segmentations from different subjects can be compared at a vertex-wise level. One benefit of this surface injection technique is that it can deal with many types of topological defects that can be present in the automated segmentation by enforcing a smoothness in the deformation that ignores holes and handles, as has been previously suggested (Khan et al., 2008).

The vertex-wise correspondence in the meshes for each subcortical ROI across subjects enables the quantification of the disease related atrophy or hypertrophy on the surface of the ROI. The deformed surfaces may lie inside (atrophy) or outside (hypertrophy) of the prototype surface. To achieve this we used a signed closest point distance metric computed at each vertex on the prototype surface to that on the target surface. At each node, *a*∈ ${\hat{S}}_{i}$, *m*∈ *S*, we find the dot-product of the displacement from prototype to the deformed injected surface, $\vec{d}$_*m, a*_ and the surface normal on the average, $\vec{n}$_*m*_. The normal distance, *d^norm^*(*m, a*) = $\vec{d}$_*m, a*_ · $\vec{n}$_*m*_ is negative or positive when the deformed surface is respectively inward or outward relative to the average, effectively an indication of the deformation.

### 2.3. Statistical analysis

In this section we describe the methods for the statistical analysis of the volume data and surface displacement features with the aim to find the group level differences in the data. Volume difference would suggest a global morphological alteration whereas surface displacement difference would suggest a localized shape change as a disease effect. The statistical tests for the surface displacement data were performed using both the rigidly and affinely pre-registered surfaces.

#### 2.3.1. Volume: group difference

The volume measurements from the surfaces generated from segmentation labels were tested for the statistical differences between the PD and HC groups. A two-tailed *t*-test was performed between the data from the two groups. The significance level was maintained at *p* < 0.05 for all tests. The test was repeated for both *UBC* and *PPMI* cohorts.

#### 2.3.2. Surface displacement data: group difference

The surface displacement data as described in the Section 2.2.5 provided a signed distance value at each vertex on the prototype surface for individual structures. The surfaces for each target subject were in vertex-to-vertex correspondence enabling a direct comparison of the displacement data. A vertex-wise comparison of group difference across the cohort between the patient and healthy control groups provided insight into the spatially-localized, disease-related alteration in shape of the subcortical structures.

A linear model was fit to regress out the effect of variation in age and gender effects from the SD data. The SD data were kept as the outcome variable and the age and gender were used as predictors in the model. The residuals from the linear model fit were used in the statistical analysis to test for the effect of disease on the data. Vertex wise group difference analysis was conducted using SurfStat software (Worsley et al., 1996), which employs Random Field Theory to correct for multiple comparisons (Worsley et al., 2004). The vertex wise comparison and cluster-forming thresholds were set at *p* < 0.05. The contrast between the two groups was evaluated as *HC*—*PD*, where a positive *t*-value suggested atrophy in patients in comparison to the healthy controls and *vice versa*.

#### 2.3.3. Relationship with clinical score

The surface displacement data were then tested for their potential to predict the UPDRS scores in both the studies.

The surface displacement data were rearranged into a 1-dimensional vector for each structure. These displacement vectors for all structures were then concatenated into a long column vector for each subject. In order to avoid the curse of dimensionality (very large dimensional data in a small sample size), dimensionality reduction was performed using Principal Component Analysis (PCA) decomposition. Sufficient number of PCs were retained to account for 95% of the variability in the data. The PC loadings were tested for the potential to predict the clinical scores in a linear model via linear regression. A leave-one-out procedure was conducted where each patients' score was predicted based on the model fit to the data from the remainder of the patient group. The PC loadings were the predictors and the clinical score were the response variables. A 2-D scatter plot (e.g., **Figure 6**) between the predicted vs. the actual score was obtained and a least squares line was fit to check for statistically significant relationship between the two. The coefficients of the linear model and their statistics are reported.

The UPDRS score in the *UBC* and *PPMI* cohorts was tested for association to the surface displacement data. We tested the UPDRS scores to be normally distributed via a lilleofors test (Lilliefors, 1967). The data rejected the null hypothesis of “not normally distributed.”

## 3. Results

**Figure 3:**
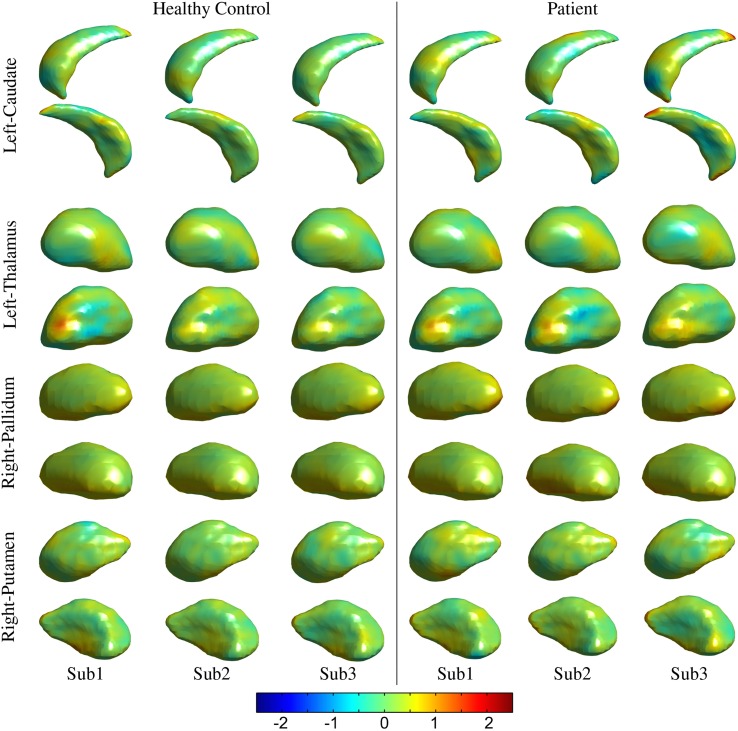
**Visualization for the quality control of the surface displacement data**. Data for three representative subjects from the healthy control and patients groups, respectively have been presented from the *UBC* dataset for left thalamus and caudate and right pallidum and putamen structures. Color (legend) represents surface displacement from the prototype surface.

The processed MRI data provided segmentation of subcortical structures for the caudate, putamen, thalamus, and pallidum in the left and right hemispheres (Figure 2). The segmentation labels for the anatomical structures were thoroughly checked for accuracy via visualization of surface outlines overlayed on the MRI slices (Figure 2). In our data, all subjects in the two cohorts were found to have acceptable (surface outline following structure boundary) and accurate segmentation labels, and were retained for subsequent analysis. Similarly, the surface displacement computed for each structure was visualized to check for presence of inaccuracies (extreme displacement values) due to registration errors. As an example, visualizations for three subjects in the patient and control groups are presented for the caudate and pallidum (right), and putamen and thalamus (left) structures (Figure 3).

**Figure 4:**
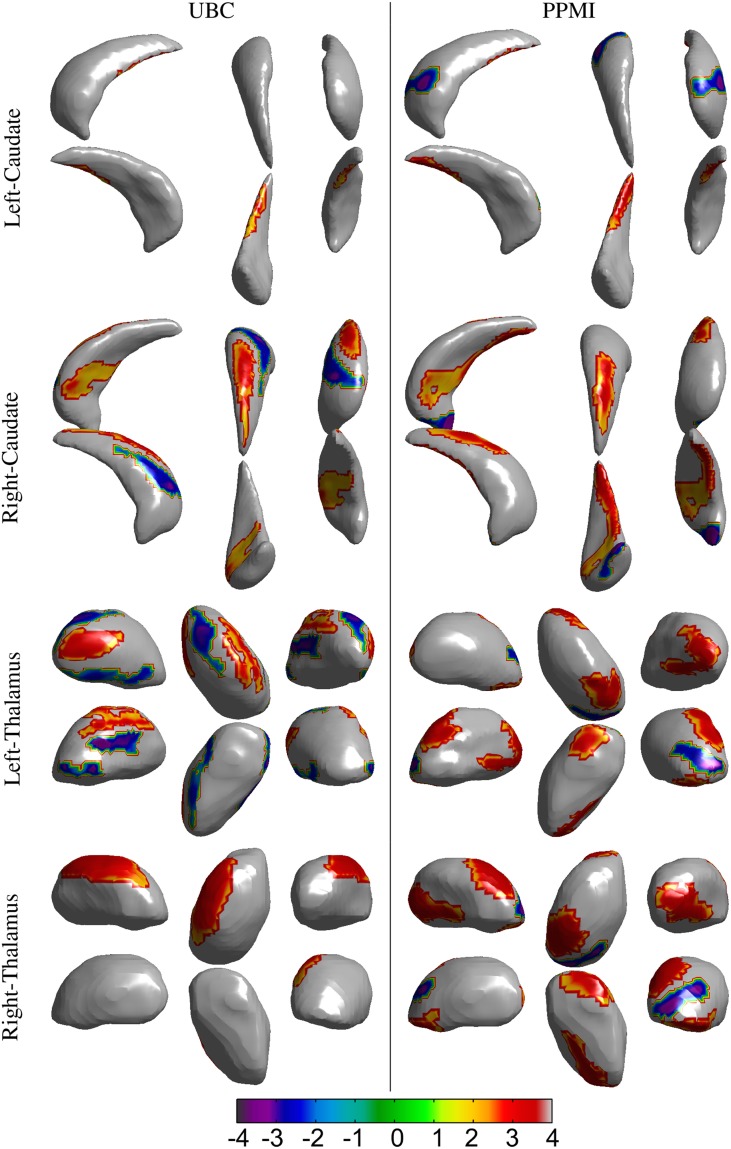
**Results for the vertex-wise group difference analysis of the surface displacement data for the left and right, caudate and thalamus structures in the *UBC* and *PPMI* datasets**. The colored patches represent the *t*-values in the areas with statistically significant (*p* < 0.05) difference between the patient and healthy control group. Gray colored area had no statistically significant difference between the groups. Positive *t*-value represents lower value in the patient group.

**Figure 5:**
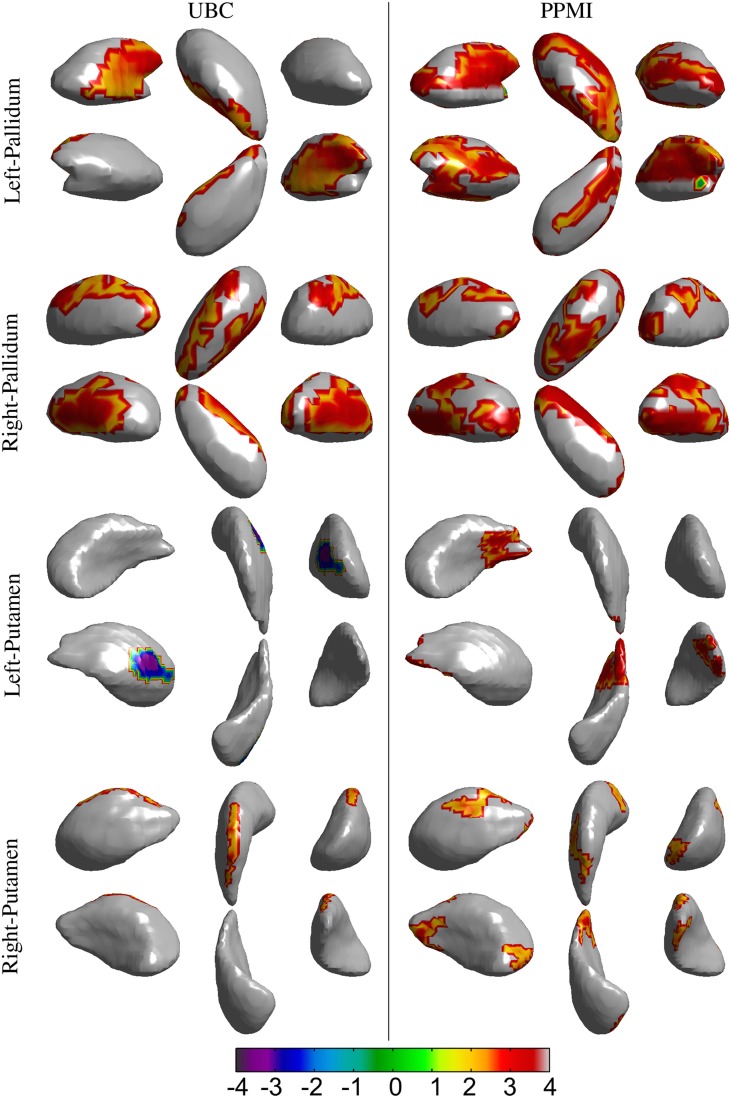
**Results for the vertex-wise group difference analysis of the surface displacement data for left and right, pallidum and putamen structures in the *UBC* and *PPMI* datasets**. The colored patches represent the *t*-values in the areas with statistically significant (*p* < 0.05) difference between the patient and healthy control group. Gray colored area had no statistically significant difference between the groups. Positive *t*-value represents lower value in the patient group.

The volumes of structures were significantly different (*p* < 0.05) for the right thalamus (*p* = 0.034, *t* = 2.15), right putamen (*p* = 0.041, *t* = 2.07), and left thalamus (*p* = 0.035, *t* = 2.14) in the *UBC* dataset. In contrast, volumes in the *PPMI* dataset did not present a statistically significant difference for all structures (Table 3). In the rigid pre-registration case, the vertex-wise group analysis did not show any statistically significant difference. However, in the affine pre-registration case, the analysis of the surface displacement data found vertex clusters with significant difference in the displacements between patient and control groups (Figures 4, 5). As the rigid pre-registration failed to highlight the significant differences between the CN and PD groups, we present the results from only the affine case in the rest of this paper. Disease-related shape changes in subcortical structures were widespread. Table 4 presents the top 2 clusters ordered by the residuals of the model fit with their number of vertices, average *t*-value, and *p*-value for each structure.

**Table 3:** **Results for the analysis of group level difference in the volume of the subcortical structures between the participants in the Parkinson's disease and healthy control groups**.

	**UBC**		**PPMI**	
**Structure**	***p*-value**	***t*-stat**	***p*-value**	***t*-stat**
L-thal	0.035^*^	2.14	0.406	0.83
R-thal	0.034^*^	2.15	0.180	1.35
L-caud	0.347	−0.95	0.262	−1.12
R-caud	0.092	−1.70	0.053	−1.94
L-pall	0.581	−0.55	0.227	−1.21
R-pall	0.606	0.52	0.945	0.07
L-put	0.126	1.54	0.959	−0.05
R-put	0.041^*^	2.07	0.400	0.84

Statistical significance at p < 0.05 marked by asterisk (^*^).

**Table 4:** **Results for the vertex-wise group difference in the surface displacement (affine pre-registered) between Parkinson's disease patients [*n* = 55 (*UBC*), *n* = 189 (*PPMI*)] and healthy controls [*n* = 54 (*UBC*), *n* = 137 (*PPMI*)] for 8 subcortical structures**.

**Structure** **(Total no. of vertices)**	**UBC PD**					**PPMI**				
	**No. of vertices**	***p*-value**	***t*-value**	**HC group**	**PD group**	**No. of vertices**	***p*-value**	***t*-value**	**HC group**	**PD group**
L-thal (2212)										
	145	0.001^*^	2.73	0.15±0.05	0.04±0.06	159	0.000^*^	3.21	0.18±0.04	0.11±0.04
	111	0.008^*^	3.06	0.09±0.05	−0.08±0.05	152	0.001^*^	3.21	0.06±0.05	−0.02±0.04
R-thal (2146)										
	244	0.003^*^	3.32	0.57±0.03	0.46±0.04	185	0.000^*^	3.27	0.48±0.05	0.43±0.04
	68	0.132	−2.53	0.11±0.06	0.28±0.06	166	0.003^*^	3.47	0.31±0.04	0.23±0.04
L-caud (2096)										
	62	0.023^*^	2.49	0.13±0.04	0.02±0.03	88	0.008^*^	3.08	0.02±0.06	−0.07±0.05
	37	0.102^*^	−2.69	−0.04±0.01	0.03±0.02	110	0.019^*^	−2.80	0.15±0.03	0.23±0.03
R-caud (2116)										
	170	0.003^*^	2.84	0.09±0.08	−0.11±0.06	306	0.000^*^	2.75	0.11±0.05	0.04±0.06
	176	0.004^*^	−2.69	0.17±0.04	0.32±0.03	152	0.004^*^	2.88	−0.12±0.07	−0.22±0.07
L-pall (522)										
	115	0.001^*^	2.70	0.60±0.03	0.55±0.03	270	0.000^*^	3.01	0.44±0.04	0.42±0.04
	14	0.086	2.58	0.33±0.03	0.28±0.02	4	0.555	−2.13	0.19±0.02	0.23±0.00
R-pall (468)										
	63	0.001^*^	2.59	0.33±0.03	0.31±0.01	147	0.000^*^	3.02	0.50±0.04	0.48±0.05
	95	0.001^*^	2.92	0.44±0.02	0.41±0.02	29	0.011^*^	3.08	0.30±0.01	0.28±0.01
L-put (1996)										
	92	0.002^*^	−2.94	−0.09±0.03	−0.03±0.03	157	0.000^*^	3.19	0.28±0.07	0.21±0.06
	51	0.097	−3.00	−0.07±0.03	0.06±0.02	67	0.032	2.51	0.10±0.02	0.05±0.03
R-put (1974)										
	75	0.008^*^	2.48	0.15±0.03	0.08±0.03	73	0.002^*^	2.58	0.30±0.07	0.26±0.07
	55	0.103	−2.65	0.07±0.02	0.13±0.03	90	0.012	2.45	0.11±0.05	0.07±0.04

Data presented for top 2 vertex clusters and their corresponding mean value for the t-statistic, p-value and the mean ± standard-deviation of the surface displacement in the HC and PD group. Structures and clusters with statistically significant (p < 0.05) difference marked with an asterisk (^*^).

Spatial clusters with statistically significant difference in the surface displacement feature in the two groups were found on all the structures. Two key observations appear from the statistical comparisons: (1) Structures with net-inward deformation for the PD group (+ve *t*-value, right putamen, right, and left pallidum) and (2) structures with both net-inward and outward deformation for the PD group (−ve and +ve *t*-value, left thalamus, right, and left caudate). Additionally, similar spatial locations with difference of same (+ve or −ve *t*-value) nature were observed in the two datasets (e.g., right and left pallidum). The clinical score prediction experiment with the data from all structures used simultaneously as a single column vector was not able to predict the UPDRS scores with the statistical significance (*p* > 0.5).

## 4. Discussion

Analysis of local shape change in comparison to global measures of morphology highlights the importance of examining spatially-localized alterations as a disease-related effect. In an extrinsic approach, the features extracted from the deformation fields rely on the choice of template to which individual images/surfaces are registered. These features can then be compared between groups for effect of a disease or clinical intervention. We computed the surface displacement metric as the signed closest point distance at each vertex of the surface model to quantify the net surface deformation. Surface displacement data (affine pre-registered) showed very strong differences between the two groups for all 4 structures (Table 4, Figures 4, 5). Such differences were observed in *UBC* and the *PPMI* datasets, where patches of vertex-clusters were present throughout the surface (Figures 4, 5). Both atrophy (positive *t*-value) and hypertrophy (negative *t*-value) were present in the patient group, suggesting compensatory alteration in shape within the same structure.

It is interesting to note that the volume of the subcortical structures did not show statistically significant difference for any of the structures in the *PPMI* dataset (Table 3). In contrast, many clusters with statistically significant (*p* < 0.05) difference in the surface displacement metric were observed in the vertex-wise group difference analysis (Table 4, Figures 4, 5). Surface displacement being a local, sensitive measure of shape change is able to present group level differences. In contrast, the gross measurement of the structural volume is shown to not yield sensitivity to disease changes. This finding is consistent with previous observations (McKeown et al., 2008) where volume did not show any difference between the disease and control group, but a shape feature was able to show statistically significant results.

### 4.1. Technical strengths of the proposed analysis approach

We applied an automated registration based segmentation approach (FS + LDDMM) which has been validated to provide accurate segmentation labels (Section 2.2.2) (Khan et al., 2008). The application of the automated method with multiple templates is expected to yield accurate segmentation of the subcortical ROI, unaffected from potential variability due to a manual labeling procedure. This segmentation method has already been shown to provide high quality segmentation for segmentation of caudate, thalamus, putamen, and hippocampus (average dice coefficient = 0.85). Another validation study in the context of segmentation of pediatric MRI images found the dice coefficients of 0.89 for thalamus, 0.89 for caudate and 0.87 for putamen, thereby emphasizing accurate segmentation results (Garg et al., 2014).

The choice of templates for segmentation is known to impact segmentation quality (Garg et al., 2014). In our study, we used templates (*n* = 6) from the *UBC* cohort which were manually segmented by a neuro-anatomy expert. The templates do differ from the data in the *PPMI* cohort, which, in itself, being a collection of data acquired at different sites, has inherent inhomogeneities due to scanner characteristics and acquisition protocols. In order to ensure good quality segmentation of the data in the two cohorts we performed thorough segmentation quality check as explained in Section 2.2.3. All the subjects in the two cohorts were found to have accurate segmentation labels, and hence were retained for subsequent analysis. This prevented the errors in segmentation from propagating into subsequent processing and analysis steps. Additionally, the use of an unbiased prototype (Sections 2.2.4, 2.2.5) to obtain the surface displacement metric ensures that the deformation data is free from bias toward a subset of the group. Therefore, the statistical outcomes obtained in our study represent the characteristic differences in the anatomy captured by the MRI data.

### 4.2. Strength of the shape feature

Structural MRI is a potential marker for alterations observed in PD as it can assess brain systems associated with motor and non-motor deficits. It has been shown that less sensitive measures of change in morphology such as volume presents contradictory results for putamen (e.g., Krabbe et al., 2005; Pitcher et al., 2012) vs. (Schulz et al., 1999), Ghaemi et al. (2002) and caudate atrophy (e.g., Pitcher et al., 2012 vs. Schulz et al., 1999; Ghaemi et al., 2002). Additionally, thalami in PD showed significant shape change despite no significant difference in volume (McKeown et al., 2008). Results from our study are in alignment with the previous observations where volumes of anatomical structures did not show a statistically significant change, whereas widespread shape change was observed as an effect of Parkinson's disease. Our shape analysis approach and its derived features contain both global and local shape information and show ability to capture sensitive disease related shape change in two large and independent datasets.

Previous work (McKeown et al., 2008) exploring differences in thalamic shape utilized a SPHARM representation. A recent morphometric analysis (Apostolova et al., 2010) also detected changes in basal ganglia structures between PD subjects with and without dementia. Apostolova's method focused on volume, where the radial distance—an intuitive measure of ROI thickness—was used as morphometric feature. Similarly, other work (Sterling et al., 2013) also modeled shape with a SPHARM-PDM representation and found areas with significant differences in the caudate and putamen structures. In contrast, our work quantified the shape change with a surface displacement measure at every vertex in the average surface model for each sucortical region. The high dimensional non-rigid registration algorithm (LDDMM, Beg et al., 2005) used in this study has been shown to account for the non-linearity of the anatomical shape space. The metric thus derived is able to accurately capture the shape alteration due to disease. Using this metric, we were able to spatially localize the areas with significant disease related alteration.

We found both similarities as well as differences in the anatomical location of shape changes between the two cohorts. The observed differences can be attributed to the difference in scanners, image acquisition protocols, inhomogeneities due to multi-center data collection in the PPMI cohort and differences in clinical features. Nonetheless, there are very encouraging overlapping changes in the PD groups in the two cohorts, mainly in sensorimotor and limbic areas and we will preliminarily discuss the functional implications of these most robust changes: The globus pallidus shows the most widespread changes with several areas of atrophy bilaterally in both cohorts; in the two datasets, areas of atrophy overlap along the medial aspect of the globus pallidus internus, most likely in areas implied in motor function (Obeso et al., 2015). In the caudate, atrophy is present along the dorso(medial) tail of the caudate on the right, affecting areas belonging to sensorimotor, frontostriatal circuits (Redgrave et al., 2010). On the left, fewer changes are observed, there is some overlap for atrophy along the ventral tail of the caudate, again likely implying sensorimotor circuits. In the left putamen, we found mostly hypertrophy in the UBC cohort but atrophy in the PPMI cohort without clear areas of overlap between the groups. On the right, areas of atrophy overlap in the dorsal area of the posterior putamen, which is associated with sensorimotor function (Redgrave et al., 2010). Both areas of hypertrophy and atrophy were observed in the thalamus, atrophy with partial overlap between the two groups is found in mediodorsal aspects of the thalami bilaterally. The mediodorsal thalamus is part of the limbic circuit and has been implied in depression in PD (Cardoso et al., 2009; Li et al., 2010). The shape feature computed in our study presents the potential to detect the differences between PD and control groups. The discriminant function that can be developed from this work could then be applied in longitudinal studies to see if it is able to detect alterations in subjects in the prodromal phase of PD.

### 4.3. Shape differences and clinical association

**Figure 6:**
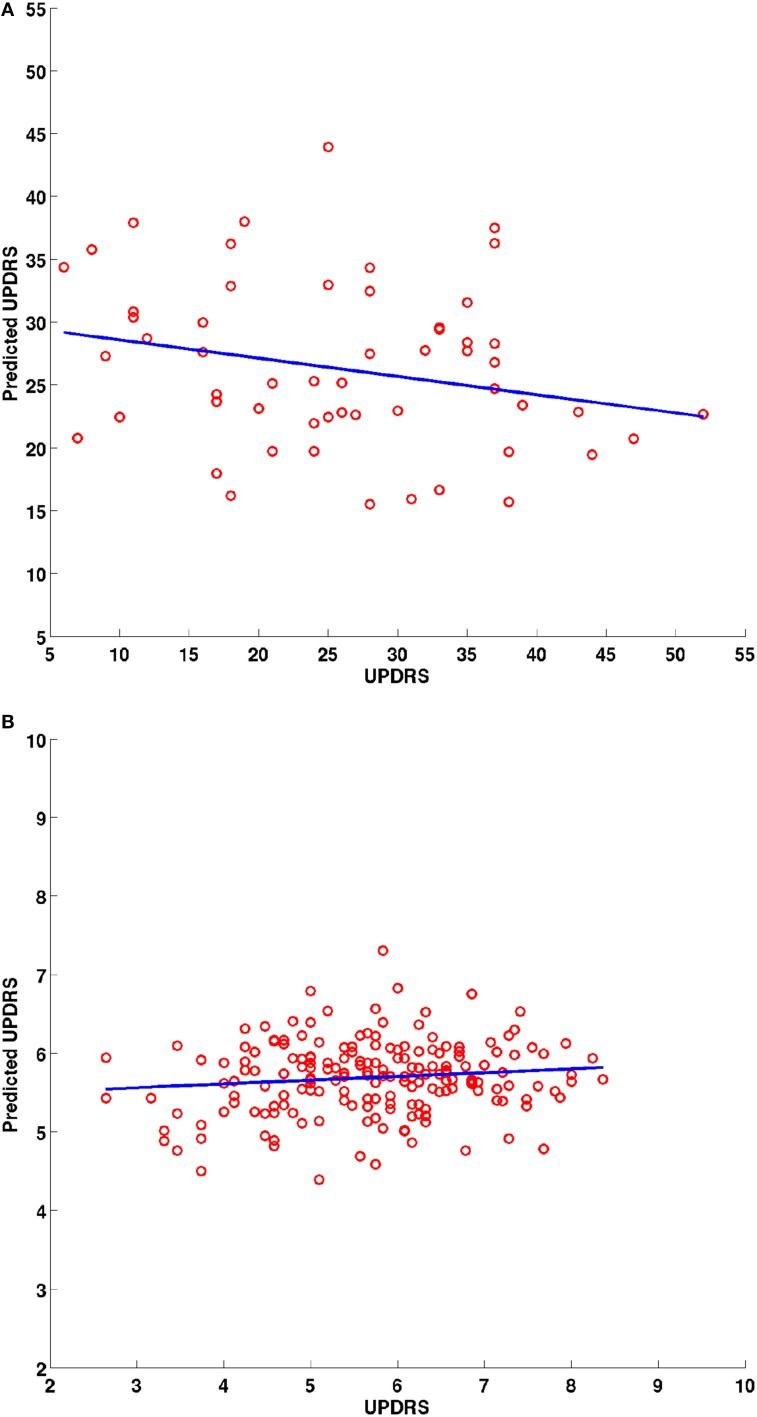
**Scatter plots for the predicted and actual clinical scores from the leave one out prediction experiment using the PC loadings from the PCA decomposition of surface displacements where data from all structures was combined**. Experimental data from the **(A)** *UBC* dataset and **(B)** *PPMI* dataset. Blue line is the least squares fit between the predicted and actual clinical score values.

The surface displacement shape feature was not able to predict the UPDRS motor score (Figure 6). The UPDRS motor score is a fairly crude clinical measurement as it combines different features of PD such as tremor and postural instability which have differing anatomical bases and this might partially explain why we did not find a correlation of the UPDRS motor score with shape changes. In order to identify correlations of shape changes with clinical indices, future studies should examine motor subscores for rigidity, bradykinesia, tremor and axial stability separately, assess mood, cognitive and reward function, control for more affected side and handedness and match groups closely for age and gender.

### 4.4. Limitation and conclusions

In our study, we considered the pre-registration of the binary segmentations using rigid and affine transformations, respectively (see Section 2.2.5). The surface displacement feature computed from the rigid registered data did not show a statistically significant difference between the groups (HC vs. PD), whereas, the affine registered data showed spatial clusters with significant difference (Table 4, Figures 4, 5). This suggests that the scale variability among the target binary segmentations has a confounding effect on the CN vs. PD group differences, and the diseases related changes are likely smaller than the variability in the scale of the structures seen across the population. The use of an affine pre-registration step between the binary segmentations removed the scale-related variability, however, in the process, it may also have removed some of the scale related changes seen due to PD. Hence, using the structures which are changing due to the disease, to further remove effects due to overall scale change across the population is likely sub-optimal. Further, the cross-talk between the normally occurring scale variability of structures in the population vs. the changes in scale due to disease (such as atrophy, or hypertrophy) likely affects all shape analysis methods that rely on surface deformation based morphometry. This observation highlights the need for shape assessment methods to deal with scale-related variability in the population better. As a suggestion, perhaps accounting for scale variability using a feature that does not change with disease such as the cranial vault instead of the binary segmentations can be investigated.

We applied volumetric registration method to obtain the mappings between the source and target binary images. As the binary images carry shape information at the boundary pixels and the pixels in the interior contain minimal or no information regarding shape of the structures, such methods are expected to provide registration accuracy and shape information, at par with surface registration methods. However, a direct comparison of the two sets of methods is beyond the focus of the current work and forms a scope for future investigation. Additionally, in order to be used as a biomarker in the clinical setting, the method needs to be tested further on much larger and independent data cohorts for its ability to predict clinical features and the ability to detect changes in the prodromal stages of the disease.

In conclusion, we present a first study to investigate the change in shape in Parkinson's disease tested on a large publicly available dataset (*PPMI*) and validated on an independently acquired dataset at *UBC*. Our results suggest that systemic changes in the shape of subcortical structures (caudate, pallidum, putamen and thalamus) can be non-invasively assessed in PD *in vivo*. The surface displacement feature encodes the spatially localized shape information. In this study, we have been able to highlight regions on the surface of subcortical structures that show changes in PD. The automated method presented in the study provides a new avenue to assess the progress of the neuro-degenerative processes. Further validation using data from larger cohorts is needed to assess the predictive capability of this method.

### Conflict of interest statement

The authors declare that the research was conducted in the absence of any commercial or financial relationships that could be construed as a potential conflict of interest.
